# Comparison of Shear Bond Strength of Three Types of Glass Ionomer Cements Containing Hydroxyapatite Nanoparticles to Deep and Superficial Dentin

**DOI:** 10.30476/DENTJODS.2019.77762.0

**Published:** 2020-06

**Authors:** Farahnaz Sharafeddin, Ali Asghar Alavi, Saba Siabani, Mina Safari

**Affiliations:** 1 Dept. of Operative Dentistry, Biomaterials Research Center, School of Dentistry, Shiraz University of Medial Sciences, Shiraz, Iran; 2 Postgraduate Student, Dept. of Operative Dentistry, School of Dentistry, Shiraz University of Medical Sciences, Shiraz, Iran

**Keywords:** Glass ionomer cements, Hydroxyapatites, Nanoparticles, Shear strength

## Abstract

**Statement of the Problem:**

The clinical success of glass ionomer cement (GIC) restorations depends on the strength of its bonding to dentin, yet the bond strength of nanohydroxyapatite (nHAp) added GIC to dentin needs to be investigated.

**Purpose:**

This study aimed to assess if the type of GIC containing nHAp and dentin depth could affect the shear bond strength (SBS).

**Materials and Method:**

In this experimental study, 60 freshly extracted intact third molars were randomly divided into two main groups of flat occlusal dentin with different cuts as superficial (S); just below the dentinoenamel junction (DEJ) and deep (D); 2mm below DEJ. After conditioning with 20% polyacrylic acid, each group were randomly assigned to the tested GIC (n=10) subgroups as (1) Fuji IX Extra+nHAp, (2) Fuji II LC+nHAp and (3) Zirconomer+nHAp. Plastic tubes were placed on the pre-treated surfaces and filled with one of the GIC, then stored in an incubator at 37 oC and 100% humidity for 24hr. The specimens were thermocycled at5/55 oC for 500 cycles and subjected to SBS test using a universal testing machine (1 mm/min). The data analyzed by Mann-Whitney and Kruskal-Wallis test (*p*< 0.05).

**Results:**

The means of SBS of Fuji II LC+nHAp was significantly higher than Fuji IX+nHAp and Zirconomer+nHAp both in superficial and deep dentin (*p*< 0.05). The means of SBS of Fuji IX Extra+nHAp and Zirconomer+nHAp subgroups in superficial dentin were higher than deep dentin, this differences was statistically significant (*p*= 0.0001 and *p*= 0.009, respectively).

**Conclusion:**

It can conclude that SBS was influenced by type of GIC and depth of dentin.

## Introduction

Glass ionomer cement (GIC) is widely used in clinical dentistry due to its unique properties which include low coefficient of thermal expansion, fluoride release, good biocompatibility and chemical adhesion [ 1 - 2 ]. Despite these attractive features, GICs have some disadvantages such as poor mechanical properties that limited their use in stress-bearing areas [ 1 , 3 - 4 ]. Since GICs were introduced, in order to improve its physical and mechanical properties and to make it more suitable for clinical use, GIC has undergone several formula changes. These changes led to production of resin modified glass ionomer (RMGI) and zirconia reinforced glass ionomer (Zirconomer), but sufficient enhancements in mechanical and chemical properties have not yet been achieved [ 4 - 6 ].

The research for more biocompatible material headed to the use of hydroxyapatite (HAp) as a biocompatible strengthening material; it has great biocompatibility and a composition similar to dental apatite, which is the main component of the tooth structure [ 1 , 7 - 9 ]. Therefore, it has attracted much interest as biomaterial filler for use in dental materials to improve the mechanical and chemical properties. Nowadays, HAp is manufactured in many forms as required for the certain applications, as nanohydroxyapatite (nHAp) with appropriate morphology, stoichiometry and purity stimulated great interest in dental material scientific researches [ 3 , 9 - 10 ]. According to the result of previous studies, it seems that incorporation of 5 wt. % nHAp improved the mechanical properties of conventional and RMGI [ 11 - 15 ].

Adhesive ability of restorative materials to tooth structure is an important factor in current restorative technique and it has been cited that the most important advantage of GIC is its chemical adhesion to enamel and dentin. The nHAp is soluble in acidic solution so that calcium ions may be extracted from the surface of the nHAp during mixing with polyacrylic liquid. The reaction mechanism that is accomplished between nHAp and GIC might be similar to the mechanism of adhesion of GIC to dentin where the interaction of apatite found in the tooth structure with the polyacrylic acid produce polyacrylate ions that form strong ionic bonds [ 10 , 14 ]. 

Various factors can influence the adhesive properties of a material, one of which is the type of dental substrate [ 11 ]. It has been reported that the dentin surface varies from tooth to tooth and due to the change in the size of dental tubules from the dentinoenamel junction (DEJ) to the pulp chamber, bonding strength, depending on the bonding site, can vary within the tooth [ 16 ]. This issue may be one of the parameters contributing to the different results in the various studies or standard deviation for each experimental group. However, there is no report to notice the effect of dentin depth in the bond properties on nHAp added GIC.

The purpose of this study was to evaluate the influence of dentin depths on shear bond strength (SBS) of three types of latest commercial GIC, including conventional, resin modified, and zirconia reinforced GIC containing nHAp. 

## Materials and Method

In this experimental study, three commercial available GIC, including a resin modified, a zirconia reinforced and a conventional GIC, and nHAp particles were used. A list of experimental materials in this study and their compositions was shown in Table 1. 

**Table 1:** Composition of the materials used in the study

Material	Manufacturer	Composition
Fuji IX GP Extra ^TM^	GC Corporation, Japan	Aluminosilicate glasses
		Polyacrylic acid powder
		Polybasic carboxylic acid
Fuji II LC	GC Corporation, Japan	Powder: Alumino-fluoro-silicate glass, Urethanedimethacrylate, Camphor Quinone
		Liquid: Polymer acrylic acid, Distill water, 2-hydroxyethylmethacrylate (HEMA)
Zirconomer Improved	Shofu Inc., Japan	Powder: Alumino-fluoro-silicate glass,
		Zirconium oxide, Tartaric acid
		Liquid: Polyacrylic acid
		Deionized water
Nano hydroxyapatite	Sigma-Aldrich, USA	Calcium hydroxyphosphate hydroxide,
		Durapatite, Hydroxyapatite
Cavity conditioner	GC Corporation, Japan	Polyacrylic acid (20%), water, aluminum chloride hydrate
Varnish	Kimia , Iran	Copal, Ethanol

First the nHAp powder was weighed carefully by a digital scale (AND; GR+360, Japan) and added to glass powders to achieve the 5 wt. % of nHAp in glass powders. In order to obtain a homogenous distribution of particles, powders were mixed initially by hand, then were transferred into specific capsules and mixed by an amalgamator (Ultramat 2; SDI, Australia) for 20 seconds. 

Sixty freshly extracted caries-free intact human third molars were selected for this study. Residual soft tissues were removed carefully, and teeth were stored in distilled water with a 0.1% thymol as disinfectant at 4oC for one week, and then stored in distilled water at 4oC until required. Teeth were mounted at 2mm below the cementoenamel junction (CEJ), in self-polymerizing acrylic resin (Acropars, Iran) in a rectangular shap-ed epoxy resin mold (30mm×25mm×15mm) as their occlusal portion were available for bonding. The teeth were randomly divided into two groups to remove the occlusal surface at two depths; superficial (SD) and deep (DD) dentin. The occlusal surfaces of the teeth were transversally sectioned by diamond discs (D&amp;Z, Germany) under water cooling to expose the flat superficial dentin just beneath the dentinoenamel junction (DEJ)in group SD and 2mm below the central groove to expose the deep dentin in group DD [ 8 ]. The exposed dentin surface of all teeth was wet grounded with 600 grit silicon carbide abrasive papers in order to achieve homogenous surface. Then the dentin surfaces were conditioned with cavity conditioner (GC, Tokyo, Japan) using a microbrush for 10 seconds, then were rinsed by distilled water for 20 seconds, and dried by cotton pellets. The specimens in each group were divided into three subgroups (n=10) according to the type of GIC as follows. 

In subgroup SD_1_, conventional glass ionomer (Fuji IX GP Extra) containing nHAp powder was mixed with liquid on glass slab by a plastic spatula according to manufacturer's instructions (powder to liquid ratio of 3.4:1 gr) for 25 seconds. The prepared paste was placed in cylindrical plastic molds (3mm diameter and 2mm height) on center of superficial dentin specimens and a Mylar strip and a glass slab were placed on the top surface of the mold for compressing until the mixture were completely set after six minutes.

In subgroup SD_2_, RMGI (Fuji II LC) containing nHAp was used and mixed with liquid by powder to liquid of 3.2:1 gr as the same manner. The specimens were restored similar to previous group and light cured for 40 seconds to ensure a perfect setting by using an emitting diode (LED) polymerizing unit (Demi Plus; Kerr, Switzerland) at a light intensity of 1200mW/cm^2^.

In subgroup SD_3_, zirconia reinforced glass ionomer containing nHAp was used and mixed with liquid by powder to liquid of 3.6:1 gr as the same manner. The specimens were restored similar to subgroup S1and waited three minutes to complete setting.

In subgroups DD_1_, DD_2_, and DD_3_, all procedures were similar to those in subgroup SD_1_, SD_2_, and SD_3_ respectively, and cylindrical molds were placed on center of deep dentin surfaces. After setting of types of GIC, plastic molds were removed. The bonded specimens were over painted by varnish (Kimia, Iran) and then were stored in an incubator (Nuve, Turkey) at 100% humidity at 37oC for 24hr before they subjected to thermocycling [ 9 ]. The specimens were thermocycled (PC300; Vafaei, Iran) 500 cycles at 5/55oC, with a dwell time of 30 seconds and transfer time of 30 seconds between baths.

**Figure 1:**
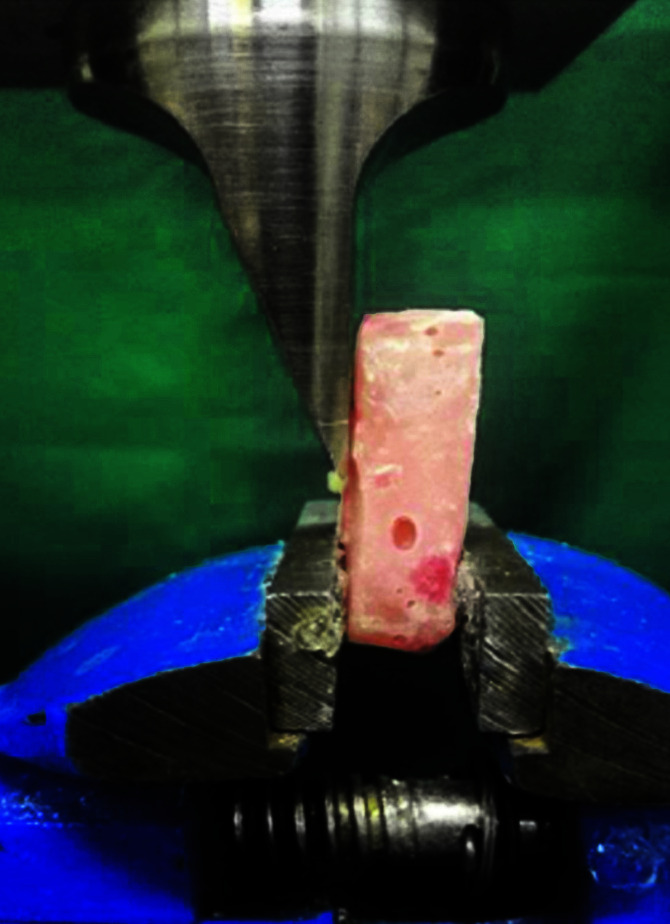
The specimen under the SBS test in the universal testing machine

The SBS of each specimen was tested using a universal testing machine (Zwick-Roell; Z020, Germany) by a steel wedge-shaped blade and crosshead speed of 1 mm/minute (Figure 1). The SBS values were calculated and reported in MPa. 

Stereomicroscopy with magnification of 40× (Bestscope, China) was used to determine the mode of failure. The stereomicroscope was performed by two calibrated postgraduate students blinded to the study.

**Figure 2:**
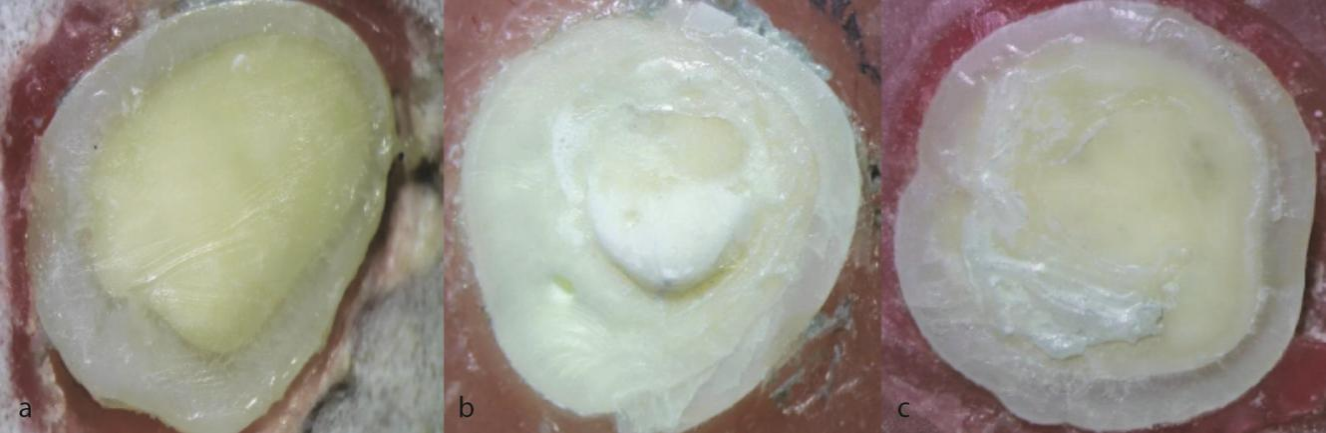
**a:** Adhesive failure (Fuji II LC+nHAp), no observable glass ionomer the dentin surface. **b:** Cohesive failure (Zirconomer Improved +nHAp), visible amounts of glass ionomer remained on the dentin surface. **c:** Mixed failure (Fuji II LC +nHAp), a mixture of both adhesive and cohesive failures

The modes of failure (Figure 2) were detected and classified as adhesive (fracture at the dentin and GIC interface), cohesive (fracture within the dentin or GIC) and mixed (fracture at the bonded interface extending into the dentin and/or GIC) [ 17 ].

### Statistical analyses

The obtained SBS values were analyzed with SPSS software (version 16), using Kruskal-Wallis H test, Dunn test, and Mann-Whitney test at significance level of *p*< 0.05.

## Results

**Figure 3:**
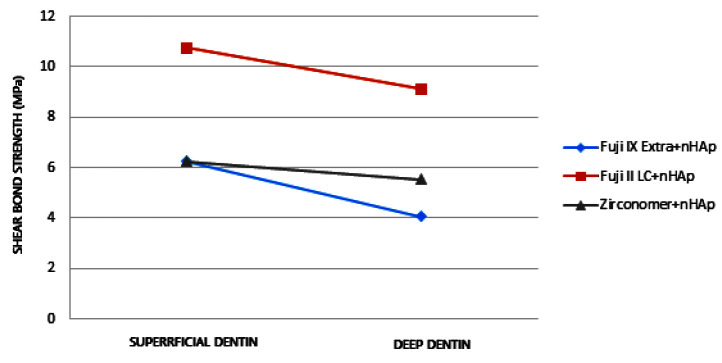
Shear bond strength of material at superficial and deep dentin groups

Table 2 reveals the mean, median values and standard deviation of SBS for experimental groups. For both superficial and deep dentin specimens, the lowest and highest means of bond strength were observed in subgroups 3 and 2, respectively (Figure 3).

**Table 2:** shear bond strength (MPa) median, mean values and standard deviation of experimental groups

GIC Subgroups (n=10)	Superficial Dentin Median (mean±SD)	Deep Dentin Median (mean±SD)	p Value*
Fuji IX Extra + nHAp	6.24 (6.21±0.72)^A^	4.06 (4.07±0.66)^A^	<0.0001
Fuji II LC + nHAp	10.75 (10.38±2.81)^B^	9.11 (9.16±1.51)^B ^	<0.481
Zirconomer Improved + nHAp	6.24 (6.25±0.65)^A^	5.53 (5.35±0.70)^A^	<0.009
** Value *	<0.0001	<0.0001	

* Kruskal-wallis H test

** Mann-Whitney U test

In each column median values with different capital letters were statistically significant (Dunn test)

Kruskal-Wallis H test showed a statistically significant difference among superficial dentin specimens; SBS value for nHAp added RMGI (subgroup SD2) was higher than the other subgroups with significant difference (*p*< 0.001). Also among deep dentin specimens, nHAp added RMGI (subgroup DD2) was higher than the other subgroups, with significant difference (*p*< 0.02). The results of Mann-Whitney test showed significant differences between subgroups SD_3_ and DD_3_ (*p*< 0.009), subgroups SD_1_ and DD_1_ (*p*< 0.000) and no significant difference between subgroups SD_2_ and DD_2_ (*p*< 0.481).

**Figure 4:**
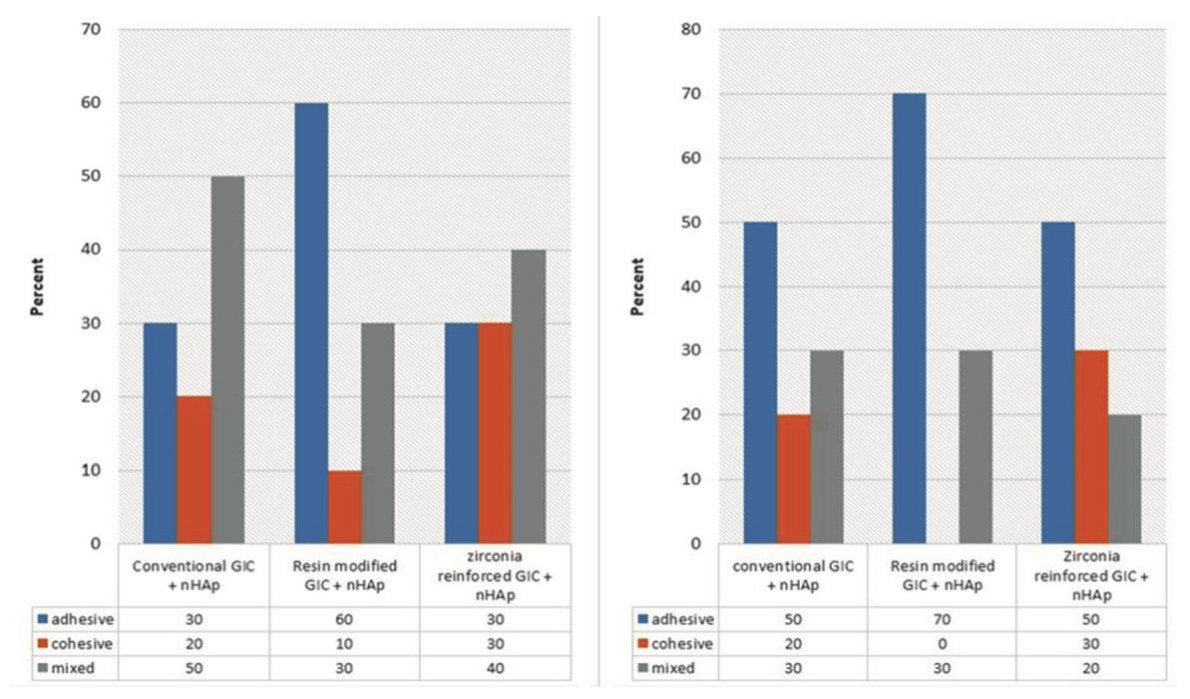
Frequency of fracture modes of three types of GIC + nHAp at superficial (left) and deep (right) dentin groups

The results of the stereomicroscope investigation at the different subgroups are shown in Figure 4. When observed under the stereomicroscope, almost all fractures were mixed and cohesive in the GIC, in subgroups 1 and 3, in superficial groups. However, adhesive failure was more frequent in deep dentin groups. Nevertheless, adhesive failure was the most common finding within subgroup 2 in both superficial and deep dentine.

## Discussion

Adhesiveness of a restoration can predict its durability. As the conventional shear and tensile tests are easy to perform, requiring minimal equipment and specimen preparation, a lot of the available data on material adhesion still comes from the macro tests, particularly regarding the GIC, which present low bond strength [ 18 - 21 ]. In the present study, the macro SBS test was used since in oral cavity, the major dislodging forces have shearing effect at interface of tooth and restoration [ 19 , 21 - 22 ]. 

The current study identified the lack of influence of dentin depth on bonding properties of three types of GIC that containing 5 wt. % of nHAp. According to our results, the SBS of RMGI plus nHAp was higher than other types of GIC with significant difference (*p*< 0.05), which was in agreement with most previous studies [ 20 - 21 , 23 - 24 ]. These results could be due to dual mechanism of adhesion in RMGI and the presence of hydroxyethyl methacrylate (HEMA), as resin component, with its superior wetting ability [ 23 ]. One study showed higher microleakage of Zirconomer compared to Fuji IX Extra and Ketac Molar [ 25 ]. Microleakage is a measure for predict the durability of restoration and is related to adhesive performance of material [ 4 ]. Conversely, the result of current study showed no significant SBS differences between Zirconomer plus nHAp and Fuji IX Extra plus nHAp subgroups to deep and superficial dentin, which might be due to addition of nHAp that improved mechanical properties of GIC and increased its adhesion to dental structures [ 12 , 26 - 27 ]. It seems that calcium ion release of nHAp reinforced the GIC by increasing the acid-base and cross-linking reactions within the GIC structure [ 10 , 12 - 13 , 27 ]. On the other hand, the formation of the strong ionic bonds between the apatite particles in GIC and calcium ions of the dental substrate enhanced the bond strength of GIC plus nHAp [ 10 , 27 - 28 ]. Although, Lucas *et al*. [ 29 ] reported that HAp would not interfere with the chemical bonding of GIC, they confirmed that addition of HAp might strengthen matrix and subsequently improve bonding between glass core and matrix.

Lin *et al*. [ 30 ] in their study, evaluated the effect of adding nanofluorapatite (nFAp) and nanofluorohydroxyapatite on fluoride release properties and bond strength of a resin modified GIC (Fuji Ortho LC), and reported an increase of fluoride release by optimum percentage of 25 wt. % but decreased SBS. The mean SBS values of nanoparticle added GIC in their study were lower than those values in present study. This difference in results could be due to using different types of RMGI. As well, lower mean SBS value in Lin *et al*. [ 30 ] study could be related to higher percentage of nHAp used which serve as defect sites and decrease mechanical properties.

A study by Kim *et al*. [ 26 ] evaluated the effect of incorporated nHAp on demineralization resistance and bond strength of Fuji II LC GIC in comparison with micro HAp. They reported addition of nHAp to GIC caused more resistant to demineralization and significant increased SBS. The mean SBS value obtained for GIC plus nHAp in their study (1.9 MPa) was significantly lower than those values in present study, which could be due to different in testing methodology. They used etched dentin as substrate and the specimens with larger bonded area, which resulted in lower SBS [ 18 , 31 ]. In addition, SBS test was done after four-week storage in pH 7.4 simulated body fluid that might have influenced the bond properties of GIC.

The obtained data of SBS values of conventional GIC in present study are almost lower than those of in Moshaverinia *et al*. [ 27 ] study that carried out with conventional GIC Fuji II containing 5 wt. % nHAp and nFAp (7 and 7.4 MPa for Fuji II plus nHAp and nFAp after 24hr storage, respectively). These lower values may be due to difference in commercial types of tested GIC, difference in depth of dentin, and the storage condition.

Another study by Lucas *et al*. [ 29 ], using a conventional GIC (Fuji XI GP) and added micro-HAp, yielded lower mean SBS to unconditioned dentin than those of the present study. This might be due to the application of cavity conditioner and employing nano size of HAp in present study. It has been reported that employing decreased size of HAp particles increases the bond strength between the tooth and HAp-added restorative materials [ 26 , 32 - 33 ].

Moreover, our results showed all GIC had higher SBS values in superficial dentin than those of deep dentin. However, this was not significant for RMGI. The same results were obtained by study of Tedesco *et al*. [ 16 ] that evaluated the influence of dentin depths and location on the micro SBS of high-viscosity GIC. Due to chemical bonding of GIC, it could be explained that the best performance of the GIC in superficial dentin could be related to the highest amount of calcium available in this area of dentin to interact with carboxyl groups. In addition, Yamakami *et al*. [ 34 ] found similar results in related to GIC. 

However, in this study, resin modified GIC showed the lower SBS to deep dentin than those of the superficial dentin; this difference was not statistically significant. The same results were verified by Hong *et al*. [ 35 ] that reported SBS of resin modified GIC remained unaffected in deep dentin. It was also demonstrated pulpal pressure had a stronger influence on bond strength than regional differences of substrate [ 36 ]. A possible explanation is that as bonded material become more hydrophilic, the SBS has lower sensitivity to dentin depth [ 31 ]. On the other hand, using extracted teeth in this study eliminated pulpal pressure and moisture arising from pulp chamber; therefore, it could be expected that after conditioning and drying of the dentin surface, the amount of moisture have been reduced. Thus, differences in the hydraulic conductance and moisture of deep and superficial dentin were not contributing factors. Therefore, the slight decrease in SBS of RMGI plus nHAp to deep dentin may be due to decreased amount of inter tubular dentin and subsequently decreased amount of calcium.

Pisaneschi *et al*. [ 24 ] evaluated SBS of GIC to deep and superficial dentin; they used Vidron R and ChelonFil as conventional GIC and Vitremer as light cured GIC. Specimens were thermocycled (500 cycles) and stored in distilled water for one week. They reported the higher SBS values in deep dentin, both for conventional and hybrid GIC in contrast with our results. These different findings could be related to difference in operated methodology and materials. Likewise, they utilized different protocol for obtained deep and superficial dentin, which may be lead to vary in dentin depth.

Failure analysis revealed higher adhesive failures in resin modified GIC plus nHAp than in the conventional and zirconia reinforced subgroups in both superficial and deep dentin. Furthermore, higher adhesive failures were observed in deep dentin specimens compared to those of superficial dentin. These results may be due to the fact that if an adhesive bond is weak relative to the strength of the GIC, failure would likely happen at the interface between the GIC and substrate [ 16 , 18 ]. It is also interesting to note that the incorporation of nHAp may result in a strengthened matrix and subsequently better bonding to the bulk of the glass and matrix [ 27 ]. Moreover, no direct relationship between the amount of SBS and mode of failure was observed in this study.

In Fuji IX and Zirconomer subgroups of the superficial group, most failure modes were cohesive/mixed rather than adhesive. These results are consistent with previous studies, which have reported the strength of the GIC–tooth bond is higher than the inherent strength of the material [ 27 , 29 ]. Also, this implies that the SBS between the GIC and the dentin could be greater than the present results. This type of failure has been commonly reported in previous studies for GIC. These findings may be related to the method of testing, which produced higher cohesive failures due to its heterogeneous stress distribution [ 37 ]. It cannot be neglected that under higher magnification, the incidence of mixed and cohesive failures might be increased for all testing modes [ 16 , 18 , 38 ]. 

As nHAp added Zirconomer is a new material and not many studies have been conducted on its properties; more research work is needed to be done to have a better vision of this new material. It seems appropriate to emphasize that it is difficult to compare the results of this study with those of others due to inconsistencies in the published literature and the lack of data regarding the adhesion of GIC plus nHAp to deep and superficial dentin. Therefore, to confirm these results, further studies are required in which different GIC categories to the different tooth substrates and other methods that simulate degradation of the bonding interface, including pH cycling and mechanical loading, as well long-term clinical trials, should be performed.

## Conclusion

Based on these findings and within the limitations of an *in vitro* study, it can be concluded that Fuji II LC plus nHAp yielded significantly different bond strengths to both superficial and deep dentin compared to Fuji IX GP Extra and Zirconomer plus nHAp. Bonding strength to superficial dentin was higher than that to deep dentin.
